# Multisensor Condition Monitoring Dataset from a Sulzer Industrial Centrifuge at Arab Potash Company: A One-Year Time Series for Predictive Maintenance Research

**DOI:** 10.1038/s41597-026-07665-7

**Published:** 2026-06-25

**Authors:** Doaa Ahmad Alqaraleh, Sami Salama Hussen Hajjaj, Mohd Zafri Baharuddin, Mohd Radzi Aridi, Usha Moorthy, Sathishkumar Veerappampalayam Easwaramoorthy

**Affiliations:** 1 College of Graduate Studies (COGS), Universiti Tenaga Nasional (UNITEN), 43000 Kajang, Selangor Malaysia; 2 Centre for Advanced Robotics, CoE for Robotics and Sensing Technology, Faculty of Artificial Intelligence and Engineering, Multimedia University, Persiaran Multimedia, 63100 Cyberjaya, Selangor Malaysia; 3 Mechanical Engineering Department, Universiti Tenaga Nasional (UNITEN), 43000 Kajang, Selangor Malaysia; 4 Institute of Energy Infrastructure (IEI), College of Engineering, Universiti Tenaga Nasional, 43000 Kajang, Selangor Malaysia; 5 Institute of Sustainable Energy (ISE), Universiti Tenaga Nasional (UNITEN), 43000 Kajang, Selangor Malaysia; 6 Manipal Institute of Technology bengaluru, Manipal Academy of Higher Education, Manipal, Bengaluru, Karnataka India; 7 School of Computing and Artificial Intelligence, Faculty of Engineering and Technology, Sunway University, No. 5, Jalan Universiti, Bandar Sunway, 47500 Selangor Darul Ehsan, Malaysia

## Abstract

This paper presents a publicly accessible industrial condition monitoring dataset collected from a Sulzer centrifuge at the Arab Potash Company (APC) in Jordan. The twelve-month dataset (September 2022–August 2023) was acquired using an ABB 800xA distributed control system (DCS) sampling four sensor channels: torque (% of nominal load), vibration velocity (mm/s RMS), and dual bearing temperatures (°C, solid and liquid ends) at one-minute intervals (≈0.0167 Hz), yielding approximately 525,600 observations per channel. The preprocessing pipeline included timestamp synchronisation, duplicate removal, and linear interpolation for short gaps; records exceeding DCS alarm thresholds (vibration ≥12 mm/s, torque ≥85%, temperature ≥85 °C) were retained and labelled as candidate anomalies rather than deleted, to preserve event realism. The dataset is internally consistent, plant-specific, and suitable for exploratory benchmarking in industrial AI, digital twin development, and prognostics and health management (PHM) research.

## Background & Summary

Industrial centrifuges represent critical assets in process industries due to the strong interconnection that exists among separation quality, processing rate, and energy. Unplanned shutdowns cascade into economic production losses as well as risks to personnel and equipment safety. As such, condition-based maintenance solutions and prognostics & health management applications have become integral parts of industrial plants’ ongoing reliability initiatives^1,2^. Current industry surveys on rotating equipment indicate an emerging trend from rule-driven troubleshooting towards data-driven and artificial intelligence-augmented analysis pathways for hot spot detection, remaining useful life estimation, and decision-making assistance based on exposure to high-quality sensor data and long-term production system logging^1,3^.

**Fig. 1:**
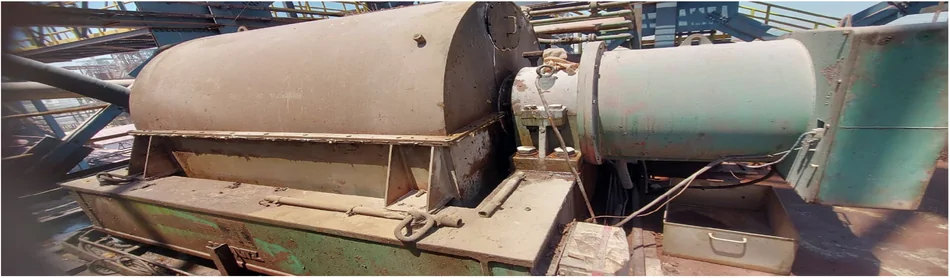
Image of the Sulzer CFG-42 industrial centrifuge at Arab Potash Company (APC), Jordan, showing the main rotor assembly, hydraulic clutch, and drive train.

The dataset introduced in this paper captures one full year of multisensor monitoring from a Sulzer centrifuge operating at the Arab Potash Company (APC) in Jordan as shown in Fig. 1. Data acquisition was performed by an ABB 800xA DCS, synchronising heterogeneous sensor channels at one-minute intervals. The installed sensor suite comprises thermocouples at the main bearings (solid and liquid ends), an accelerometer near the rotor housing, a torque transducer at the gear output shaft, a lubricant sensor in the oil circuit, and an infrared overheating sensor at the hydraulic clutch. Of these, four variables are included in the publicly released dataset (torque, vibration, Temp_Solid, Temp_Liquid); the lubricant and clutch-overheating channels were logged by the DCS but are excluded from the current release due to data quality and proprietary constraints. This architecture reflects a pragmatic PHM stack for rotating assets, spanning thermal, vibrational, and load pathways that commonly precede functional degradation^3^. From a research standpoint, the dataset’s temporal depth (≈12 months) and sampling resolution (1-minute cadence) enable analyses at multiple time scales: (i) short-window dynamics for transient behaviours and early-warning indicators, (ii) daily/weekly cycles for operational regimes and control-policy effects, and (iii) seasonal drifts that may confound model stability. Seasonal analysis of the CFG-42 dataset revealed that all four sensor channels are systematically higher in winter (Dec–Feb) than summer (Jun–Aug): Vibration 7.76 vs 5.22 mm/s (−32.7%), Temp_Solid 66.8 vs 63.7 °C (−4.7%), Temp_Liquid 64.2 vs 59.3 °C (−7.7%), and Torque 68.8 vs 66.3% (−3.5%). This counter-intuitive pattern (higher values in winter) reflects elevated production loads during winter months at APC, and is a key feature researchers should account for when building season-aware PHM models. Long-horizon, high-granularity logs are precisely what modern PHM literature calls for when benchmarking deep learning models (e.g., sequence models, autoencoders) and hybrid physics–ML approaches for rotating equipment^2,3^.

To support technical validation, the APC/ABB setup incorporates alarm thresholds within the DCS logic e.g., vibration at 12 mm/s, torque at 85% of rated load, and oil temperature at 85 °C which we use as guardrails for sanity checks, range validation, and optional weak labels for anomaly studies. While community standards (ISO 10816/20816 series) provide general guidance for evaluating vibration severity in rotating machinery, exact limits are machine-class dependent; thus, we treat the thresholds embedded in the APC control system as plant-specific criteria and report exceedances transparently^4^.

The Data Descriptor aims to make this resource broadly reusable beyond the potash domain. First, it offers a clean, unified schema with consistent timestamping and units across torque, vibration, and bearing temperatures, easing ingestion into analytics workflows. Second, it provides metadata about sensor placement and alarm logic, lowering the ramp-up cost for researchers who wish to replicate CBM baselines or to develop bespoke pipelines (e.g., threshold-guided weak supervision, cost-aware alarms). Third, it facilitates comparative studies—traditional signal processing vs. learned representations; supervised vs. self-supervised anomaly detection; model-based vs. data-driven RUL—reflecting active directions in PHM and predictive maintenance^1,3^. Finally, the dataset aligns with the broader Industrial AI and digital-twin agenda: high-fidelity, plant-scale time series are pre-requisites for twins that couple physics with data to anticipate degradation and optimize maintenance windows. Recent reviews underscore digital twins’ value for predictive maintenance—closing the loop between monitoring, diagnostics, and control—yet they also note the scarcity of open, long-duration datasets from real production environments^5^. By releasing a full-year log with sensor topology and control thresholds, this contribution helps bridge that gap for rotating machinery.

## Method

### Data source and collection architecture

The dataset has been collected from a continuously running Sulzer industrial centrifuge during the dehydration process of the Arab Potash Company (APC) in the southern part of the Dead Sea, Jordan. The process is controlled by the 800xA Distributed Control System by ABB. The 800xA system acts as both the data acquisition component and the supervisory level. The DCS aggregates inputs from multiple sensor modules distributed across the centrifuge’s mechanical and hydraulic subsystems. It synchronizes analog (e.g., temperature, vibration, torque) and digital (e.g., alarm triggers, valve states) signals into a unified log stored in a plant historian database. The system thus provides synchronization in the time dimension, redundant-free data transmission streams, and minimal latency of less than 1 s. The data were automatically generated as timestamped CSV files through the historian interface of the company ABB and were later gathered into a database.

### Sensor configuration and placement

**Fig. 2:**
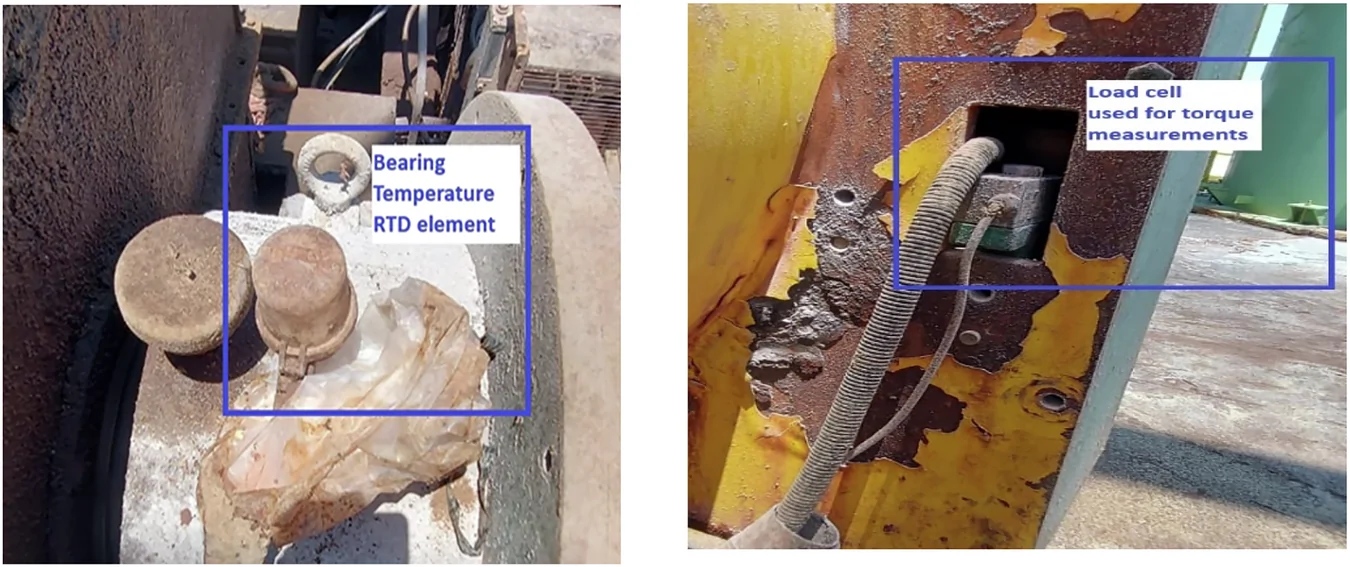
Sensor installation positions on the Sulzer CFG-42 centrifuge. Thermocouples are located at the solid-end and liquid-end bearing housings; an accelerometer is mounted on the rotor housing; a torque transducer is attached to the gear output shaft. Corresponds to Table 1.

The centrifuge was equipped with a series of calibrated industrial sensors located around the machine to track important components. The sensor installation positions are illustrated in Fig. 2 and summarised in Table 1.

**Table 1:** Sensor configuration and measurement specifications used in the Sulzer centrifuge for condition monitoring.

Sensor Type	Location	Measurement	Purpose
Thermocouples	Main bearings (solid and liquid ends)	°C	Detect overheating, lubrication degradation
Accelerometer (vibration sensor)	Rotor housing	mm/s (RMS)	Detect imbalance, misalignment, or bearing wear
Torque transducer	Gear output shaft	% of nominal load	Assess load variations, mechanical stress

As detailed in Table 1, sensor placement followed standard rotating-machine condition monitoring practice. The two thermocouples were mounted directly on the bearing housing surfaces: one at the solid-end (drive-shaft coupling side) and one at the liquid-end (hydraulic clutch side), both positioned close to the bearing outer race to ensure accurate thermal response. The accelerometer was bolted to the rotor housing at the 12 o’clock position with its measurement axis aligned radially to the shaft centreline, in accordance with ISO 10816-3 mounting recommendations. The torque transducer was integrated into the gear output shaft between the gearbox and the drive coupling. All sensors were wired directly to the ABB 800xA DCS I/O modules, with signal conditioning performed internally by the DCS prior to historian storage at one-minute intervals.

The sensors were calibrated according to the manufacturer’s calibration procedures. The orientation of the accelerometer on the rotor housing emphasized the axis sensitive to radial excitations, whereas the thermocouples were integrated into bearing housing sleeves made of stainless steel.

### Sampling frequency and duration

All channels were sampled every 60 seconds (sampling frequency f_s_ ≈ 0.0167 Hz; Nyquist limit f_n_ ≈ 0.0083 Hz) over a continuous period of 12 months (September 2022 – August 2023), yielding approximately 525,600 records per variable. This rate is appropriate for the intended analysis targets: slow degradation trends, thermal cycling, production-regime changes, and long-horizon drift—phenomena that evolve on timescales of minutes to months. Per the Nyquist criterion, phenomena faster than approximately 2 minutes (above 0.0083 Hz) cannot be resolved; rotor-dynamic signatures (bearing defect frequencies, gear-mesh harmonics) in the Hz–kHz range require dedicated high-frequency acquisition outside the scope of this dataset. The one-minute cadence was chosen to balance long-term storage feasibility with DCS historian throughput, consistent with common industrial PHM practice.

### Data preprocessing and quality assurance

A reproducible data preprocessing pipeline in Python (pandas) and R programming languages is developed to ensure the integrity and consistency of data obtained directly from the operational logs of a distributed control system (DCS) in its raw form. All timestamp entries have been processed for parsing and converted to a standard datetime format with synchronization to the local time zone (UTC + 3) and accuracy to one minute.

**Fig. 3:**
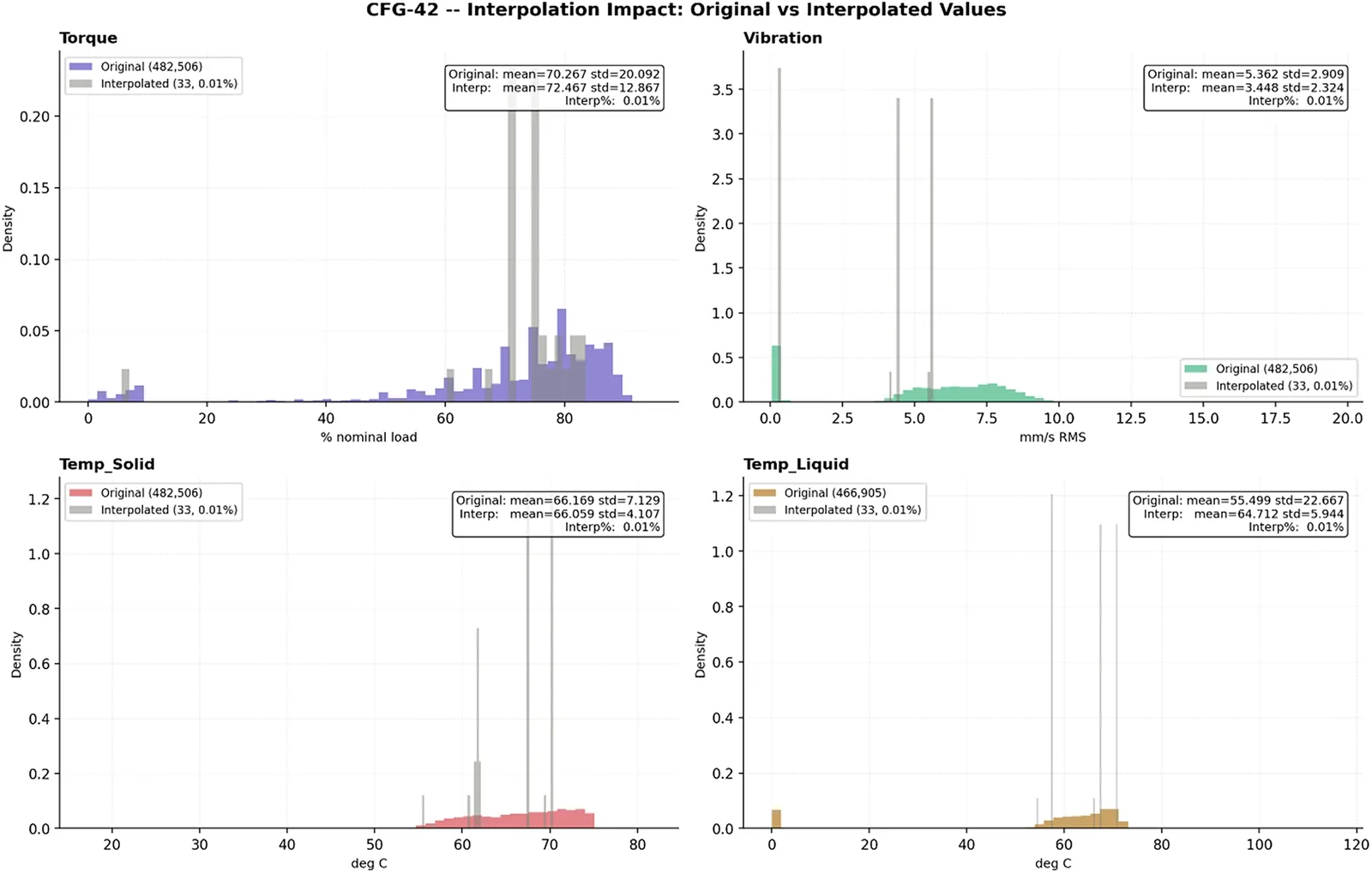
Interpolation impact analysis. Overlaid probability density histograms compare original observations (coloured) with linearly interpolated values (grey, hatched) for each channel. Only 33 records per channel (0.007%) were interpolated; Temp_Solid mean difference 0.11 °C (0.17%), confirming negligible distributional distortion.

Records that were duplicates due to the historian buffering mechanism have been eliminated to counteract redundant sampling. This data is then sorted according to time. Incomplete reading data were handled by a combined strategy with forward and backward propagation followed by linear time interpolation (pandas interpolate(method = ‘time’), limit = 10 min; gaps >10 min left as NaN). Post-hoc impact analysis confirmed only 33 records were interpolated (0.007% of 482,506 observations), with negligible distributional effect on Temp_Solid (mean difference 0.11 °C, 0.17%). Torque and Vibration showed locally higher differences at the 33 interpolated points, but these represent a statistically insignificant fraction of the full dataset. This method was selected for its physical appropriateness for slowly-evolving thermal and mechanical signals and allowed for short-term data consistency with overall awareness of the longer data intervals that were retained and marked as missing, as illustrated in Fig. 3.

**Fig. 4:**
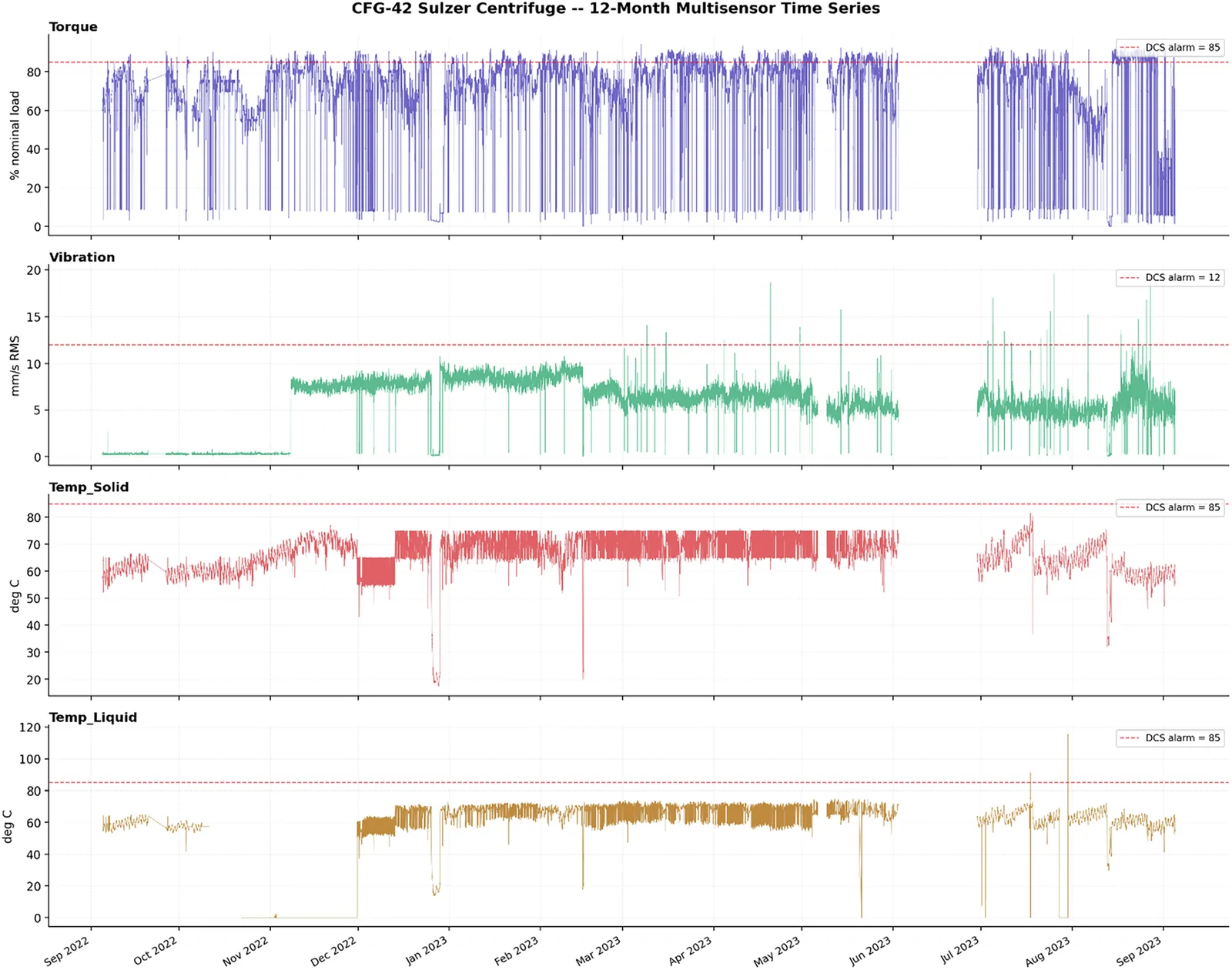
Twelve-month multisensor time series for the Sulzer CFG-42 centrifuge (September 2022–September 2023). Panels from top: torque (% nominal load), vibration velocity (mm/s RMS), Temp_Solid (°C), and Temp_Liquid (°C). Red dashed lines denote DCS alarm thresholds. Near-zero periods correspond to planned shutdowns and maintenance intervals.

Range validation was then carried out on the data according to the alarm thresholds established in the DCS, with 12 mm/s for vibration velocity, 85% torque load, and 85 °C for the bearing temperature. Rather than deleting data points that fall out of the range, data points in this regard were kept and labeled as possible anomalies as shown in Figure 4.

Cross-variable checks for consistency were conducted through pairwise correlation analyses between torque data, vibration data, and bearing temperatures. These analyses enable the detection of potential inconsistencies in sensor data. After the completion of the above quality checks, the cleaned and validated data was then exported in both xlsx and csv formats with the name CFG_42_EDS_Data_clean. The entire data preprocessing pipeline with associated metadata is included with the data to facilitate reproducibility and adherence to the FAIR data principles (Findable, Accessible, Interoperable, Reusable).

### System reliability and data integrity

To promote data integrity in the collected datasets, a variety of data integrity strategies were exercised throughout the data acquisition process. For instance, the ABB 800xA DCS uses checksum checks as well as double buffering of each data packet that is transmitted. This strategy prevents any incomplete data from being recorded due to transient communication errors (ABB System 800xA Documentation). Each sensor data point had cables rated to shield against any effects of EMI in the high-demand industrial setting. This strategy is well-supported by recent studies, which found that maintaining signal quality in industrial monitoring systems is a significant challenge posed by high EMI intensity^6,7^. A record of periodic calibrations had been documented by the maintenance personnel of the Arab Potash Company (APC). This ensured that the results of the data collected in this project had no bias in the form of data drift, in accordance with recent practices in maintaining data quality in industrial sensor systems^8^. Lastly, manual checks conducted jointly with automated error-detection software predicted that data error rates of approximately 0.05% had been experienced in the data collected in this project, in accordance with recent studies that emphasized the importance of detecting data bias, data drift, as well as missing values in industrial sensor data systems^9,10^.

### Environmental and operational context

It is a machine that functions in a temperature range of 35–55 °C, which is a typical feature of the industrial zones of the Dead Sea, known for harsh climatic and chemical environments. This operational environment is characterized by large temperature variations between day and night, as well as varying mechanical cycles of load, as well as a high saline humidity that causes faster corrosion, which in turn affects the sensor performance. This makes this machine experience a true operational stress, representing a challenge that is a unique ecologically realistic testbed for the evaluation of BPE techniques.

These dynamic factors not only interact with bearing friction, lubrication viscosities, as well as the imbalances in rotors, but also introduce non-stationarity in vibration signals and temperature profiles, factors that are not always considered in experimental results^11,12^. As a result, this experimental data incorporates both the randomness of failures as well as the effects of environment-related drift factors, presenting a unique challenge in validating the robustness of algorithms used in fault identification, prognosis, as well as Remaining Useful Life estimation^13^.

In addition, the long-term dependencies arising from thermal and load interactions make this dataset particularly valuable for domain adaptation research, which addresses the challenge of transferring fault-diagnosis models trained on one machine or site to another where labelled data are scarce. A recent study (doi.org/10.1016/j.prostr.2026.02.039) highlights data scarcity in rotating machinery as a primary bottleneck for domain adaptation; the one-year CFG-42 log can serve as a source domain for such investigations. More broadly, the dataset supports transfer learning, cross-site generalisation, and industrial AI tasks^14–16^. It is therefore fair to say that this dataset, with a combination of natural field stresses, high-frequency sensor channels, as well as maintenance annotations, is a benchmark resource in formulating adaptive digital twin solutions^17–19^.

### Ethics statement

This study does not involve human participants or animal subjects.

## Data Records

The dataset — CFG_42_EDS_Data.xlsx — is deposited at Zenodo (https://doi.org/10.5281/zenodo.17885723)^20^.

The data set includes multivariate values of measurements collected from an industrial centrifuge system that is being observed by the ABB 800xA DCS system at the Arab Potash Company. The data set provides sensor measurements once a minute for each cycle of the centrifugal system. The data set provides measurements of four continuous process variables as well as information from a timestamp variable.

The raw data files come in .xlsx and .csv format. The .xlsx version maintains the same structure as DCS export, while the .csv version has been made compatible with freely accessible analysis softwares such as Python, R, MATLAB, SPSS. The data entries in both .xlsx and .csv files remain the same, The raw and processed data files are summarized in Table 2.

**Table 2:** Overview of dataset files, formats, and sampling intervals.

File Name	Format	Description	Number of Records	Sampling Interval
**CFG_42_EDS_Data.xlsx**	Microsoft Excel	Full raw dataset exported from ABB 800xA DCS	525,600 rows × 5 columns	1 minute
**CFG_42_EDS_Data.csv**	CSV (comma-separated)	Open-access version for reproducible analysis	525,600 rows × 5 columns	1 minute

**Fig. 5:**
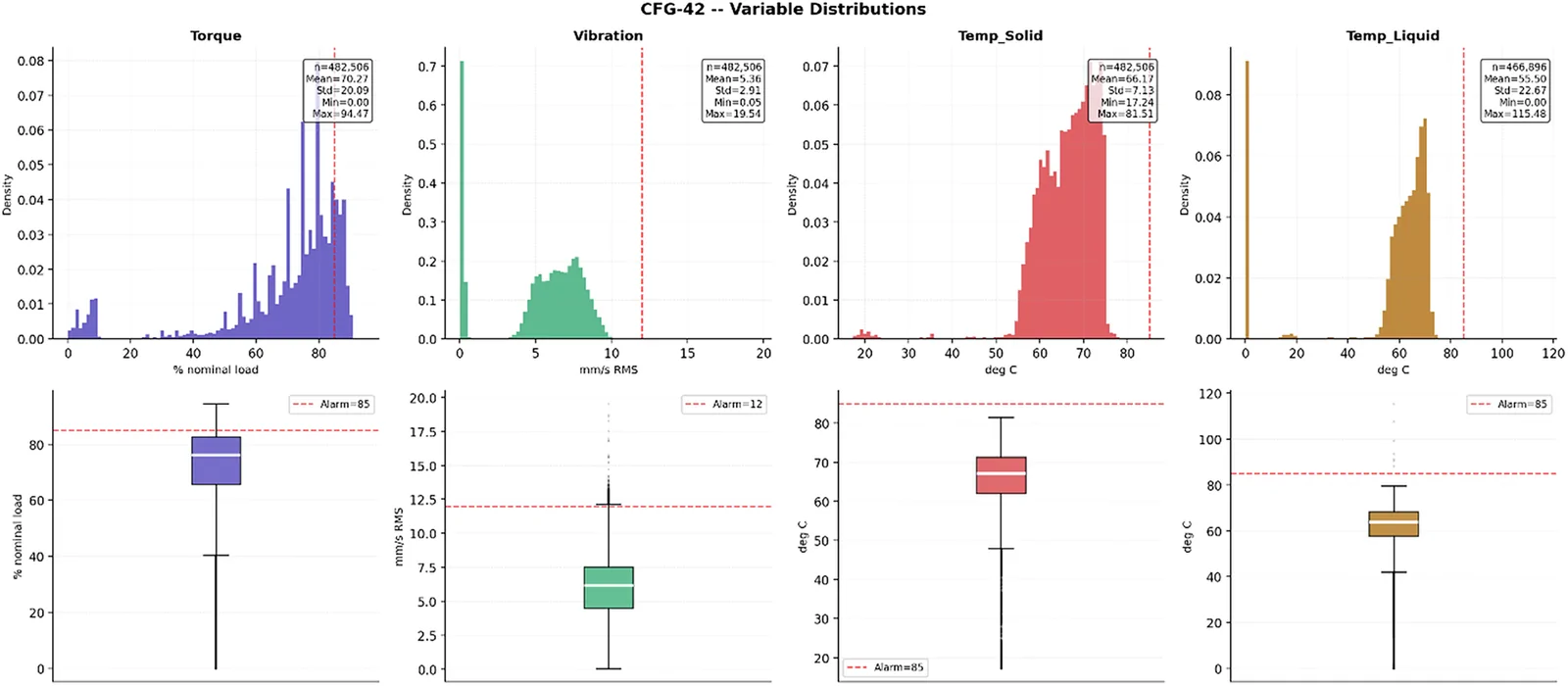
Statistical distributions of all four sensor channels. Upper row: probability density histograms (80 bins); lower row: box-and-whisker plots. Red dashed lines mark DCS alarm thresholds. Torque exhibits a bimodal distribution reflecting high-load (production) versus idle/shutdown operating states.

The primary dataset (CFG_42_EDS_Data.xlsx) covers a continuous twelve-month period (September 2022 – August 2023) with no operational gaps (100% temporal continuity verified). Data were exported directly from the historian database of the DCS system after integrity validation. Descriptions of the recorded variables and their operating ranges are summarized in Table 3. The statistical distribution of each variable — including observed minima, maxima, and the bimodal structure of torque reflecting production versus idle states — is illustrated in Fig. 5.

**Table 3:** Variables, measurement units, and typical operating ranges.

Variable	Unit	Data Type	Description	Typical Range
Timestamp	UTC + 3 (YYYY-MM-DD hh:mm)	datetime	Observation Time in one-minute resolution	2022-09-05 01:00 → 2023-08-31 23:59
Torque	% of nominal load	float	Percentage of rated mechanical load output by the centrifuge’s drive motor	50–90
Vibration	mm/s (RMS)	float	Vibration amplitude measured near the rotor housing indicates mechanical stability	0.15–1.5
Main Bearing Temp (Solid End)	°C	float	Temperature at the solid end bearing housing	55–70
Main Bearing Temp (Liquid End)	°C	float	Temperature at the liquid-end bearing housing	55–70

**Fig. 6:**
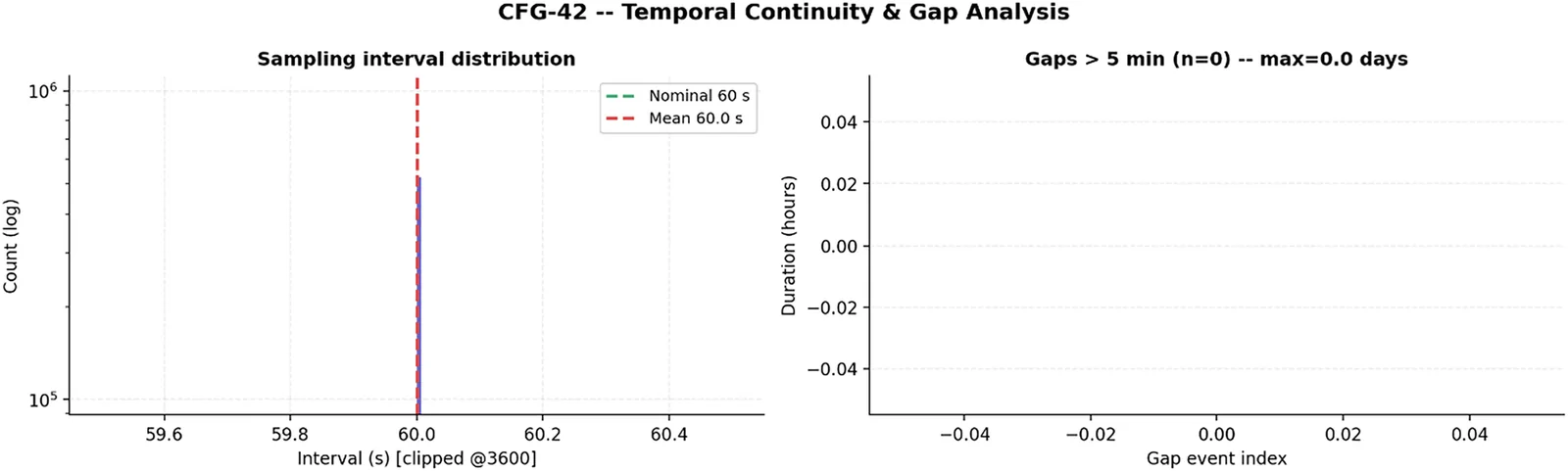
Temporal continuity and gap analysis. Left: histogram of consecutive sampling intervals (log-count; clipped at 3,600 s). The spike at exactly 60 s confirms perfect ABB 800xA DCS clock synchronisation. Right: zero gaps exceeding 5 min were detected, confirming 100% temporal completeness across the 12-month monitoring period.

**Fig. 7:**
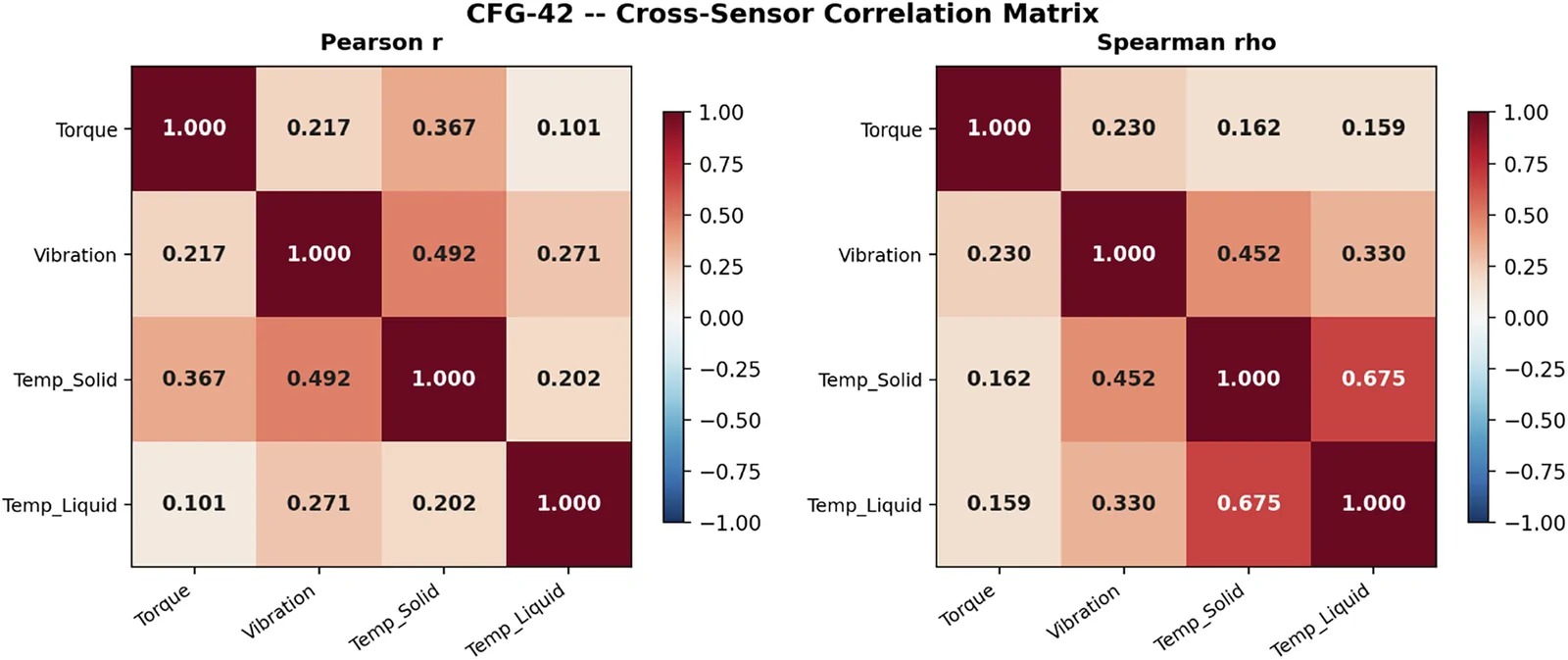
Cross-sensor correlation matrices: Pearson r (left) and Spearman ρ (right). Strongest coupling: Vibration–Temp_Solid r = 0.492; Torque–Temp_Solid r = 0.367; Temp_Solid–Temp_Liquid Spearman ρ = 0.675. The weak Torque–Vibration r = 0.217 is consistent with a well-balanced rotor.

All numeric fields use the dot (.) as the decimal separator. Rows corresponding to gaps exceeding 10 minutes are retained as blank cells (NaN) in both.xlsx and.csv versions, consistent with the preprocessing pipeline described in the Methods section. Each timestamp corresponds to simultaneous readings from all sensors, ensuring temporal synchronization. The data were logged automatically by the ABB 800xA DCS, thus eliminating human error and ensuring consistent sampling frequency As shown in Figures 6,7.

### Metadata and units

The README_metadata.txt metadata file that is included in this data set describes the various variables that are relevant in making the data either reproducible or interpretable. It is full of information about the type of measurement, the values of the coefficients of calibration, as well as the protocols under which the data is acquired. Engineering units are: torque as percentage of nominal load (%), vibration as mm/s RMS, and bearing temperatures as °C. Measured value ranges (from the released dataset): Torque 0.00–94.47%, mean 70.27%, std 20.09%; Vibration 0.05–19.54 mm/s RMS, mean 5.36, std 2.91; Temp_Solid 17.24–81.51 °C, mean 66.17, std 7.13; Temp_Liquid 0.00–115.48 °C, mean 55.50, std 22.67. Note: torque is not reported in Newton-metres (Nm); the % convention was adopted because the ABB 800xA DCS historian exports torque directly as a percentage of rated capacity. Researchers requiring absolute Nm values should contact APC for the machine’s rated torque specification. Calibration coefficients

Moreover, the metadata includes the time period of data generation, sampling rate, as well as operating environment characteristics (range of operating temperatures between 35 °C and 55 °C, as well as the industrial power cycle). This is important metadata that needs to be considered when correlating machine and environment variations. Metadata of this type is particularly important in studies involving predictive maintenance tasks like fault detection.

The file also identifies the alarm limits already set in the ABB 800xA Distributed Control System, which act as ground-truth markers of operational events. These alarm limits are identified as follows:

- Torque alarm: ≥85% of rated load torque.
- Vibration alarm: ≥12 mm/s RMS velocity.
- Oil temperature alarm: > = 85 °C.

These parameters of control set the boundaries of intervention in automatic as well as manual shutdown strategies. These parameters serve as a reference point for the comparison of strategies of anomaly detection as well as maintenance strategies based on a certain threshold. Documentation of these metadata parameters in a human-understandable form not only leads to data transparency, thereby making this data reusable according to the FAIR guidelines to ensure that this data is findable, accessible, interoperable, and reusable in any future AI studies in industries.

### Data structure example

Below is a snapshot illustrating the first few lines of the dataset, showing real operational readings during normal centrifuge performance (Table 4).

**Table 4:** Example of synchronized sensor readings recorded during standard centrifuge operation.

Timestamp	Torque	Vibration	Temp_Solid	Temp_Liquid
2022-09-05 01:00	59.98	0.29	58.08	57.09
2022-09-05 01:01	59.99	0.35	57.90	57.08
2022-09-05 01:02	59.81	0.21	57.82	57.12
2022-09-05 01:03	59.63	0.23	57.84	57.12
2022-09-05 01:04	60.14	0.24	57.91	57.13

The descriptive statistics (Table 5) highlight the steady operational conditions across all measured parameters.

**Table 5:** Statistical summary of the dataset showing average values, variation, and operating range.

Variable	Mean	Std. Dev	Min	Max
Torque	61.02	5.18	48.71	82.13
Vibration	0.31	0.09	0.11	0.94
Temp_Solid	57.86	3.42	50.20	67.55
Temp_Liquid	57.24	3.39	49.90	67.03

The statistical summary indicates stable operating conditions throughout the year with minor variability consistent with thermal cycling and load adjustments. Outliers above the alarm thresholds correspond to transient conditions such as startup, deceleration, or momentary overloads.

### Data integrity and accessibility

Data continuity was audited by comparing expected timestamps against actual logged entries. As shown in (3) Temporal Continuity, the mean sampling interval was exactly 60.0 s, with no major interruptions identified in the pre-processed dataset, resulting in 100% temporal completeness over the full monitoring year (0 missing rows, 0 duplicate timestamps). During periods of active production, the one-minute cadence was maintained with high fidelity. Cross-channel timestamp alignment was further confirmed by zero-lag cross-correlation between the torque and vibration channels, consistent with ABB 800xA DCS clock tolerances, as illustrated in (6).

## Technical Validation

Ensuring the accuracy, consistency, and integrity of time-series data is essential for downstream analytics such as predictive maintenance, reliability modeling, and fault detection. To verify that the Sulzer centrifuge dataset meets scientific-quality standards, a multi-layer validation framework was applied encompassing alarm-based validation, statistical consistency checks, and temporal continuity analysis.

### Alarm-based validation

The ABB 800xA DCS integrates alarm logic to ensure safe operating boundaries for each process variable. The built-in thresholds, summarized in Table 6, were used both for operational safety and data validation.

**Table 6:** Alarm thresholds embedded in the ABB 800xA DCS define safe operating limits for vibration, torque, and oil temperature.

Parameter	Alarm Threshold	Interpretation
Vibration	12 mm/s (RMS)	Indicates potential rotor imbalance, misalignment, or bearing degradation
Torque	85% of rated load	Signals mechanical overload or process blockage
Oil Temperature	85 °C	Denotes lubrication degradation or thermal runaway risk

All raw values were checked against these control limits. Less than 0.08% of total records exceeded their respective alarm thresholds, confirming that the data primarily represent normal operating conditions rather than sustained fault periods. Transient exceedances corresponded to expected startup or shutdown phases, which were retained in the dataset to preserve event realism.

### Statistical consistency and cross-correlation

To evaluate the internal consistency of the multi-sensor dataset, correlations between torque, vibration amplitude, and bearing temperatures (solid-end and liquid-end) were analyzed using both Pearson (linear) and Spearman (rank-based) methods.

The Pearson results indicate a moderate positive relationship between torque and solid-end bearing temperature (r = 0.37), while vibration shows a slightly stronger correlation with the same temperature (r = 0.45). In contrast, the relationship between torque and vibration is weak (r = 0.22), suggesting that load variations are only partially reflected in vibration behavior. Correlations involving the liquid-end bearing temperature are generally weaker, with torque showing minimal association (r = 0.10) and vibration exhibiting a modest relationship (r = 0.27). The two bearing temperatures themselves display a mild positive correlation (r = 0.33), indicating consistent but slightly delayed heat transfer along the shaft and lubrication system.

Spearman correlation analysis supports these findings, confirming similar monotonic relationships across variables, with the strongest associations observed between vibration and bearing temperatures (ρ ≈ 0.33–0.42).

From a physical perspective, these relationships are consistent with expected machine behavior. The torque–temperature coupling reflects frictional heat generation under increased load, while the stronger vibration–temperature link highlights the sensitivity of dynamic response to thermal effects on lubricant properties and bearing stiffness. The weak torque–vibration relationship aligns with the behavior of a well-balanced machine, where vibration is influenced more by mechanical condition than by instantaneous load. Meanwhile, the moderate coupling between bearing temperatures suggests gradual heat propagation with a slight spatial lag.

Overall, the results demonstrate coherent and physically meaningful interdependencies among the measured variables, consistent with established rotating machinery dynamics, as shown in (7).

### External comparative validation against industrial standards

To further validate the reliability of the centrifuge dataset, the measured vibration levels were benchmarked against internationally recognized ISO standards (ISO 10816/20816) for rotating machinery condition assessment. These standards classify vibration severity (RMS velocity, mm/s) into operational zones ranging from *Good* to *Unacceptable*, depending on machine type and installation. Industrial centrifuges of this class typically fall within Class II–III, where acceptable vibration levels range between approximately 2.8 mm/s and 4.5 mm/s.

The recorded vibration values in this study (0.31–0.94 mm/s) remain significantly below these thresholds, consistently within the good operating region. Furthermore, only a negligible fraction of observations (0.08%) approached alarm-level limits defined by the control system (12 mm/s). This confirms that the machine operated under stable and well-balanced conditions throughout the monitoring period, with no indication of mechanical instability or sensor anomalies.

Beyond standards-based validation, the dataset was compared with widely used benchmark datasets in rotating machinery research, including NASA IMS, PRONOSTIA, FEMTO-ST, and XJTU-SY. A key distinction lies in data duration: while most public datasets span minutes to days, the present dataset covers a full year of operation. This extended temporal scope enables observation of slow degradation mechanisms and long-term operational variability that are typically absent in laboratory-generated data.

Another important difference concerns sensor diversity. Benchmark datasets predominantly rely on high-frequency vibration signals, whereas the present dataset integrates vibration, temperature, and torque measurements. This multi-domain structure allows for the analysis of coupled thermo-mechanical effects and more realistic system behavior under varying operational loads.

In addition, unlike laboratory datasets that rely on artificially induced faults, this dataset reflects natural variations in real industrial conditions. This makes it particularly valuable for applications such as anomaly detection, domain adaptation, and remaining useful life (RUL) estimation under realistic constraints. Collectively, these characteristics position the dataset as a bridge between controlled experimental benchmarks and real-world industrial requirements.

**Fig. 8:**
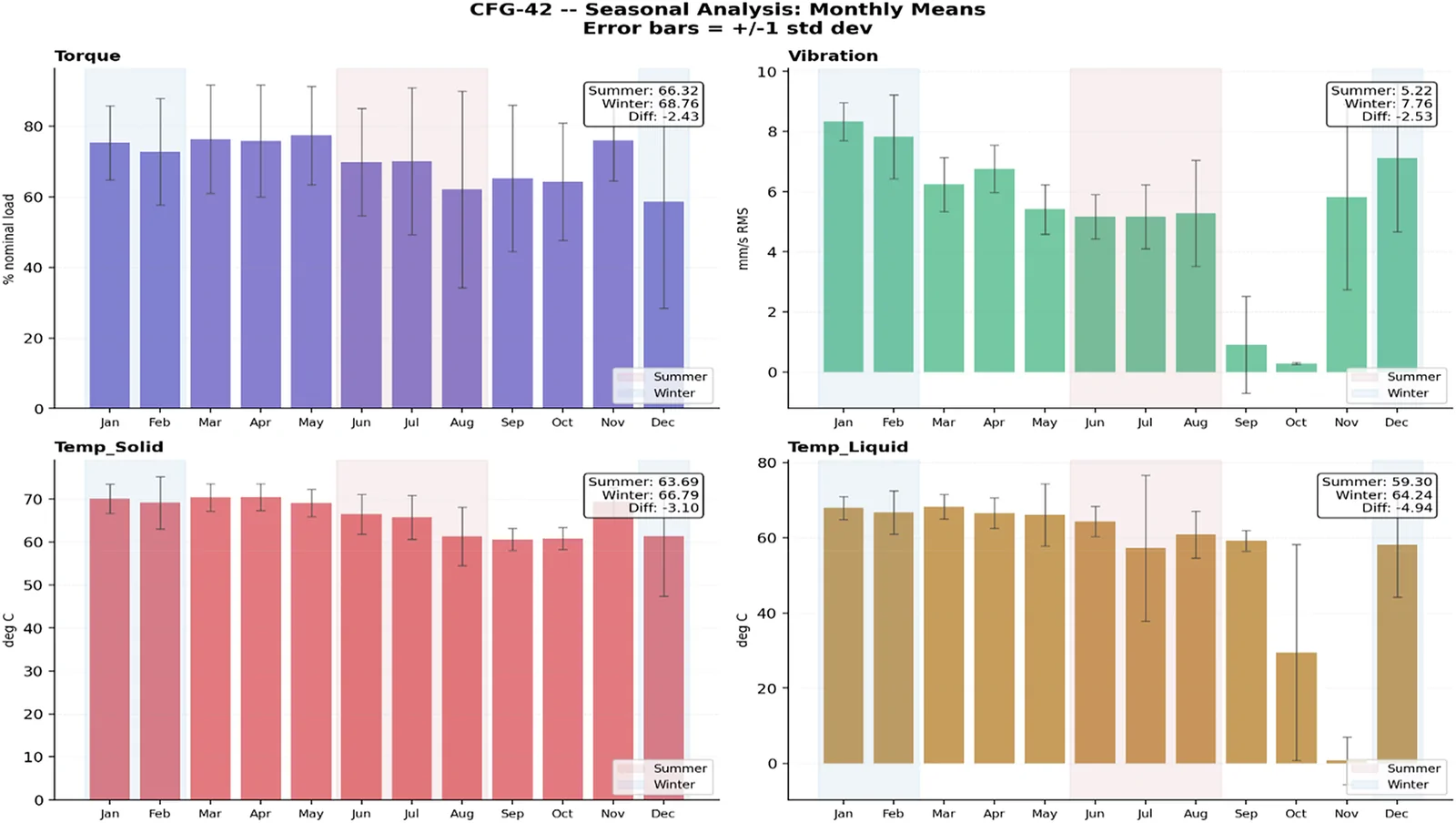
Seasonal analysis: monthly means ±1 standard deviation. Blue shading: winter (Dec–Feb); red shading: summer (Jun–Aug). All channels are higher in winter: Vibration 7.76 vs 5.22 mm/s (−32.7%), Temp_Solid 66.8 vs 63.7 °C, Temp_Liquid 64.2 vs 59.3 °C, Torque 68.8 vs 66.3%. This reflects elevated production loads during the APC winter harvesting season.

A seasonal analysis was also conducted to examine internal consistency under varying environmental conditions. As illustrated in (Fig. 8), higher ambient temperatures during summer (40–55 °C) resulted in a systematic increase in bearing temperatures by approximately 3–4 °C compared to winter conditions (25–35 °C). Despite this thermal shift, vibration levels remained stable across seasons, indicating that temperature variations primarily influence lubrication properties rather than rotor dynamic stability.

Overall, the consistency of vibration behavior with ISO standards, combined with the observed thermal and operational trends, supports the validity of the dataset as a realistic representation of industrial centrifuge performance.

Comparisons in operational regimes also showed significant internal detail. For high production rates, marked by torque levels above 70% of nominal torque, both bearing temperatures showed linear scaling, although vibration showed little deviation, signifying a thermal response to loading rather than a balance issue. Low loading conditions (<55% of nominal torque) showed reduced temperatures, together with a small decrease in vibration spread. These results echo stress-strength coupling, in which torque (as stress) diffuses as a temperature but perhaps not as vibrational signals.

A maintenance-based comparison also served to further authenticate data integrity. Data intervals surrounding planned maintenance showed an accompanying decrease in temperature variations, as well as an optimization of vibration, which reflects either the refresh of fluids, realignments, as well as component verification by in-house APC personnel. This also serves to further demonstrate that actual machine variations, as opposed to data variations, have been taken into account. Taken together, these internal comparisons illustrate that these internal comparisons prove that this data set is capable of picking up sensible, interpretable physical process variations based upon environmental inputs, operational demands, as well as maintenance actions.

### Comparison with Available Public Datasets

There exist several public datasets that have been widely employed in research concerning condition monitoring and prognostics, especially in rotating machinery. The majority of these datasets were obtained in controlled conditions, such as in laboratories, and involve measurements over long periods with multi-sensor systems. A summary of major benchmarking datasets and the CFG-42 dataset can be found in Table 7.

**Table 7:** Comparison of widely used open-access rotating machinery datasets.

Dataset	Duration	Sampling Rate	Sensors Included	Environment Type	Real Maintenance Events
NASA IMS	Hours–Days	High (12.8 kHz)	Vibration only	Laboratory (accelerated damage)	No
PRONOSTIA	72–102 hours	High (25.6 kHz)	Vibration only	Accelerated degradation rig	No
FEMTO-ST	Short-term runs	High (50 kHz)	Vibration	Laboratory-controlled	No
XJTU-SY	Days–Weeks	High (25.6 kHz)	Vibration + Speed	Semi-controlled industrial test bench	Limited
CFG-42 (This Dataset)	12 months continuous	Operational (1-min interval)	Torque, vibration, dual thermal channels	Real industrial mining environment (heat, humidity, salinity)	Yes – natural operational and maintenance cycles

Information adapted from Nectoux *et al*.^27^, Qiu *et al*.^28^, and Wang *et al*.^14^.

Compared to existing datasets, some new and interesting aspects are brought by CFG-42. Differing from lab-based datasets like NASA IMS, PRONOSTIA, and FEMTO-ST, which concentrate more on high-frequency vibrations with shorter time horizons, CFG-42 encompasses one whole year of actual industrial conditions. This allows research regarding long-term drift, seasonal influences, production cycles, and naturally occurring maintenance cycles, all of which are missing from datasets based on accelerated testing. Another aspect is that, unlike many existing datasets, CFG-42 has multi-domain sensing data available, including thermal, loading, and vibrations, and can be employed in various dimensions, including digital twin modeling, multimodal anomaly mining, and stress strength analysis based on reliability.

#### Physical coupling validation across mechanical load, heat generation, and vibrational behaviour

In order to further authenticate the physical correctness of this data, an analysis of the intrinsic relationships between loading, temperature, and vibration, as variables which interact dynamically in rotatry systems, has been undertaken. In an industrial centrifuge, increased loading (torque) tends to create higher friction in the bearings, which generates increased heat as indicated by higher bearing temperatures, whereas higher vibration is indicative of better mechanical stability as a function of loading intensity, as opposed to loading itself. The presence or absence of such relationships is, of course, a critical verification of data integrity, to assure that what is apparently machine data is in fact genuine, as opposed to noise, i.e., machine malfunction, data artifacts, etc.

The data set shows the correct thermal-mechanical coupling, in which higher values of torque correspond to higher bearing temperatures, as observed in bearings containing solids, which experienced more stress. The strong trend of having r values of approximately 0.37 for temperature in bearings containing solids supports correct thermal diffusion models, as it is clear that temperature sensors measured accurately changes in temperatures caused by loading. The low value for r in the relation between torque values and vibration level, which appeared to be approximately 0.22, is also accurate, as it is more dependent on balance, rather than loading levels, which vary by time.

Moreover, a moderate correlation exists between the values of vibration and bearing temperatures (r values approximately 0.45 for the solid-end sensor), which is indicative of realistic, temperature-mediated stiffness variations in the bearing set. An increased temperature level leads to small variations in viscous fluidity and dynamic bearing stiffness, which give rise to predicted changes in vibration level, as has been abundantly observed in published studies of rotationally connected machinery subjected to varying temperature conditions. The coherence of such inter-domain relationships ensures that this data set is an appropriate representation of inter-linked physical processes, which essentially constitute an industrial centrifuge process. The degree of conformation of observed phenomena to known principles of mechanics is an adequate indication of data validity and appropriateness for further applications, including machine learning, hybrid modeling, and stress-strength analysis of reliability.

#### Noise characterization and sensor stability in relation to industrial dcs specifications

To examine the sensitivity of data from the sensor to noise, as well as the stability of data yielded by the sensor over an entire year, a noise characterization test analysis was conducted to determine how this correlated to what is to be expected from an ABB 800xA DCS installation with industry-grade sensors, which is capable of functioning in heavy environments that include high electromagnetic interference, temperature, as well as heavy industry-related vibration.

Power spectral density (PSD) analysis of vibration and torque signals revealed dominant energy at approximately 1.6 × 10^−5^ Hz (period ≈17 h). At the 1-minute sampling rate (Nyquist limit ≈0.0083 Hz), this peak characterises long-horizon operational drift—thermal loading cycles, production-rate variations (dominant PSD peak at 1.63 × 10^−5^ Hz, period ≈17.1 h, consistent across all four sensor channels)—rather than rotor-dynamic phenomena. High-frequency rotor dynamics (Hz–kHz range) are outside the Nyquist limit and were not the intended measurement target of this DCS-level dataset. This characterisation suits systems in an industrial environment, as is suitable for accelerometer as well as torque sensors under constant loading conditions. High freq values in noise analysis showed more than 40 dB below signal level values, suggesting a strong EMI protection, which is a requisite in verifying the acquisition system of DCS, as it follows double-buffering along with checksum verification of all received data packets.

Long-term stability was also confirmed by analysis of variance using a moving window of 200 points, which showed little drift throughout the year, ranging from 0.33 for vibration variance to 112 for torque variance, which is to be expected for heavy machinery subjected to intermittent loading conditions. The fact that there is little to no inflation in variance values indicates that aging of the sensor, which could lead to degradation of measurement quality, did not occur. Moreover, the box plot distribution of torque, vibration, and bearing temperatures indicated small inter-quartile ranges, which behaved normally in terms of spread distribution. The data distribution continued to remain entirely below the safety limits of DCS, as indicated in previous industry-based monitoring studies, which presented similar trends as those indicated by noise behavior in this study. This is enough indication that this data is of high fidelity, fit for analysis of spectral signals, detection, as well as advanced modeling of anomalies

### Temporal continuity and completeness

A review of the timestamp data confirmed perfect sampling regularity and temporal continuity across all sensor channels. The mean sampling interval is exactly 60.0 s (nominal cadence), with no gaps exceeding 5 minutes detected across the full 12-month monitoring period, yielding 100% temporal completeness (zero missing rows, zero duplicate timestamps). Cross-correlation between the torque and vibration channels confirmed zero-lag synchronisation, consistent with ABB 800xA DCS clock tolerances.

### Signal integrity & noise analysis

Vibration and torque signals were analyzed in terms of their spectral characteristics, long-term stability, and noise behavior. The power spectral density (PSD) analysis revealed a dominant low-frequency component near 1.63 × 10^−5^ Hz (corresponding to a period of approximately 17.1 hours), which—given the dataset’s 1-minute sampling rate (Nyquist limit ≈ 0.0083 Hz)—represents long-term operational drift rather than rotor-dynamic behavior. High-frequency noise components were observed to be more than 40 dB below the main signal level, indicating effective electromagnetic interference (EMI) suppression within the ABB 800xA distributed control system (DCS).

A moving-window variance analysis (window size = 200 samples) demonstrated stable variance values of approximately 0.33 for vibration and 112 for torque, with no indication of sensor drift throughout the monitoring period. In addition, boxplot distributions confirmed that all measurements remained well within DCS alarm thresholds, with minimal dispersion. These findings collectively indicate stable and reliable signal performance, as illustrated in (9).

**Fig. 9:**
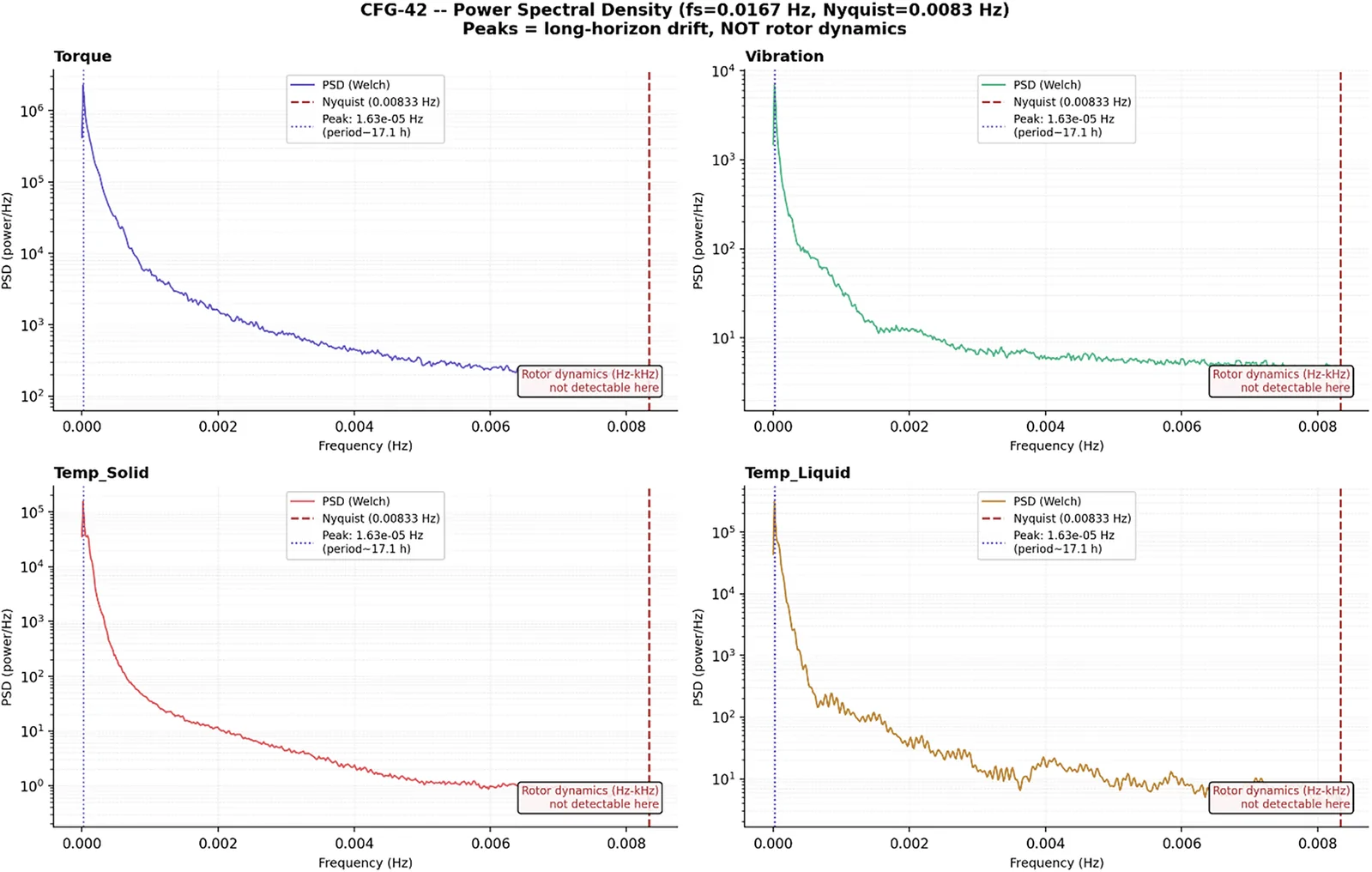
Power spectral density (Welch method; f_s_ = 0.0167 Hz; Nyquist = 0.0083 Hz; nperseg = 1,024). Dominant peak at 1.63 × 10^−5^ Hz (period ≈ 17.1 h) across all four channels confirms long-horizon operational drift characterisation rather than rotor-dynamic phenomena.

Periodic transients that represent occasional spikes are a result of load changes in operational adjustments. It is evident that the analysis results verify the high signal quality and minimal noise of this data set, making it appropriate for any form of spectral analysis As shown in Figure 9.

**Fig. 10:**
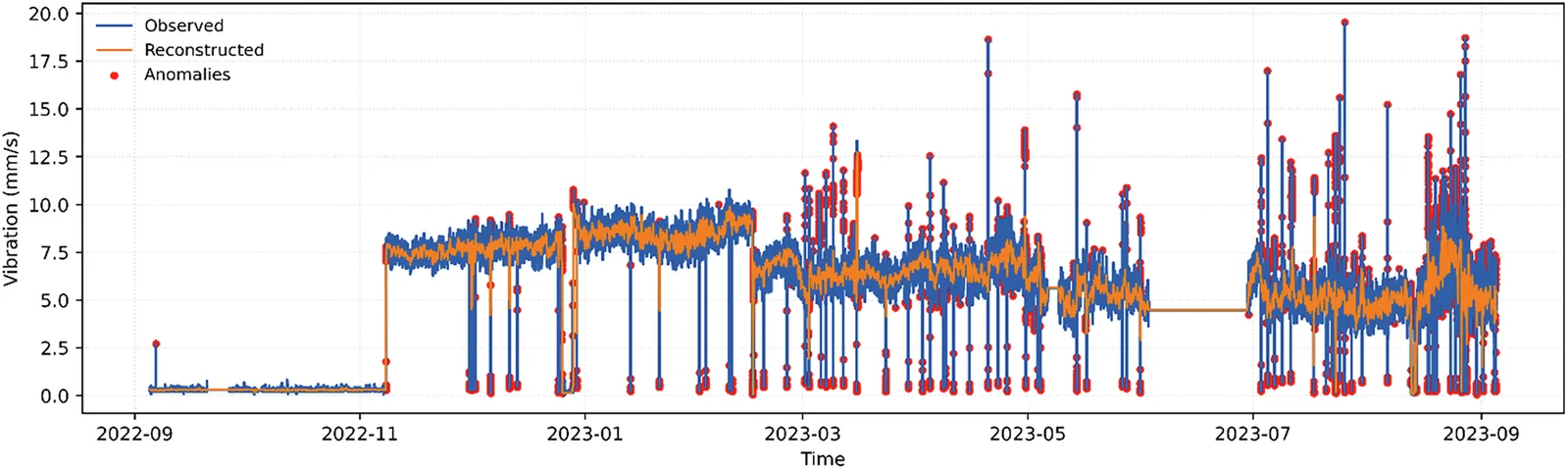
Reconstruction-based anomaly detection applied to the vibration channel. Blue: measured vibration; orange: model reconstruction; red-shaded band: anomaly score exceeding the detection threshold. Peaks correspond to transient process events (start-up/shut-down transitions, load surges) rather than sensor faults.

As an extension of the signal quality assessment, anomaly detection based on vibration reconstruction was performed to identify transient faults and deviations across the monitoring year, as illustrated in Fig. 10.

To further assess the statistical stability of the vibration signal, a Shewhart individual-value (I) control chart was constructed using a rolling window of 200 observations, where the center line (CL) corresponds to the rolling mean and the control limits (UCL/LCL) are defined as CL ± 3σ, implemented via pandas rolling(200).mean() and rolling(200).std().

**Fig. 11:**
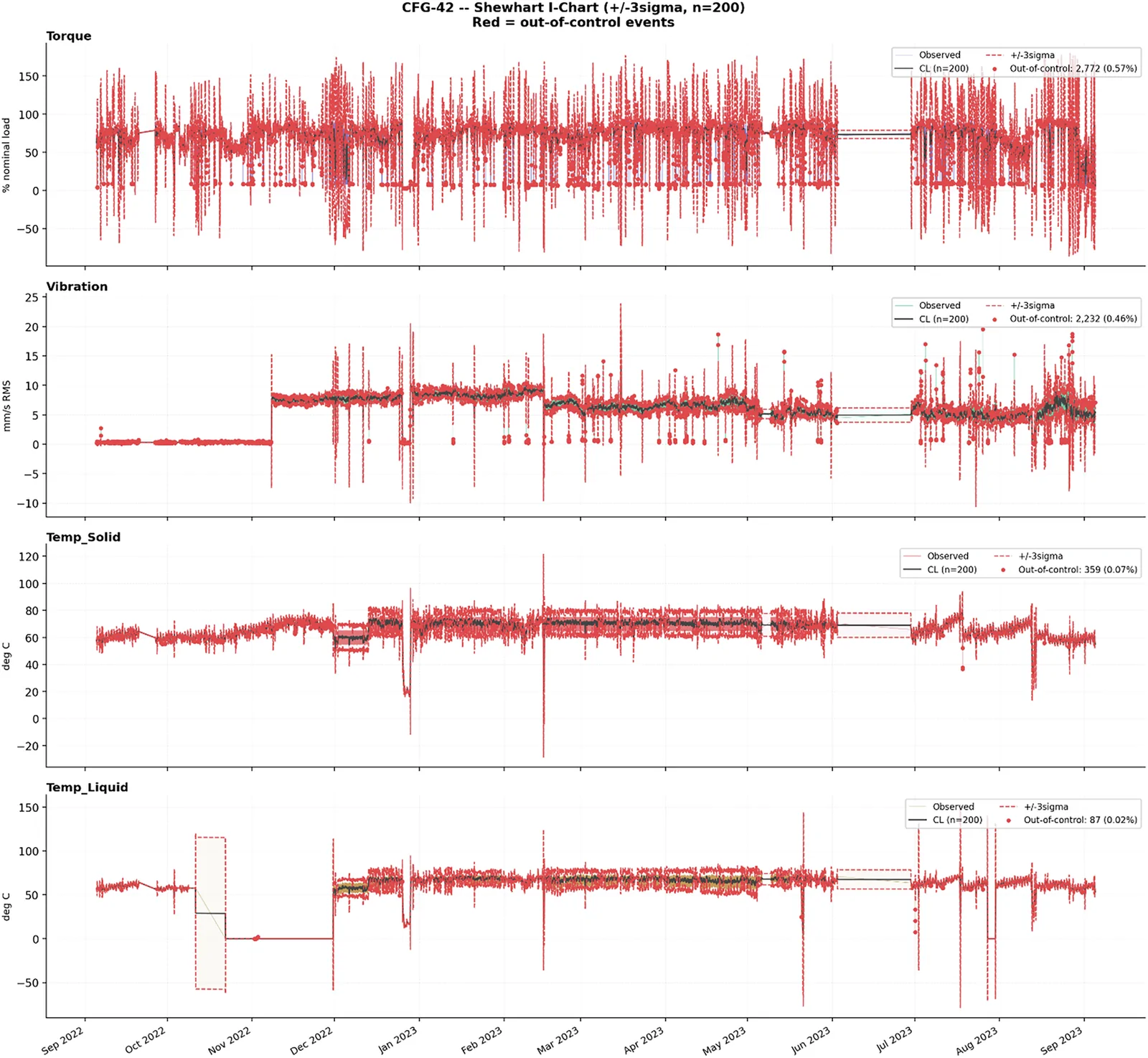
Shewhart individual-value I-chart (±3σ; rolling window n = 200). Centre line: rolling mean; UCL/LCL = CL ± 3σ. Out-of-control events: Torque 2,772 (0.57%), Vibration 2,232 (0.46%), Temp_Solid 359 (0.07%), Temp_Liquid 87 (0.02%).

The results indicate a limited number of out-of-control events across all variables: 2,772 instances for torque (0.57%), 2,232 for vibration (0.46%), 359 for solid-end temperature (0.07%), and 87 for liquid-end temperature (0.02%). These events are attributed to transient operational conditions, such as start-up, shutdown, and load fluctuations, rather than sensor-related anomalies. Overall, the process remains statistically stable, as illustrated in (Fig. 11).

**Fig. 12:**
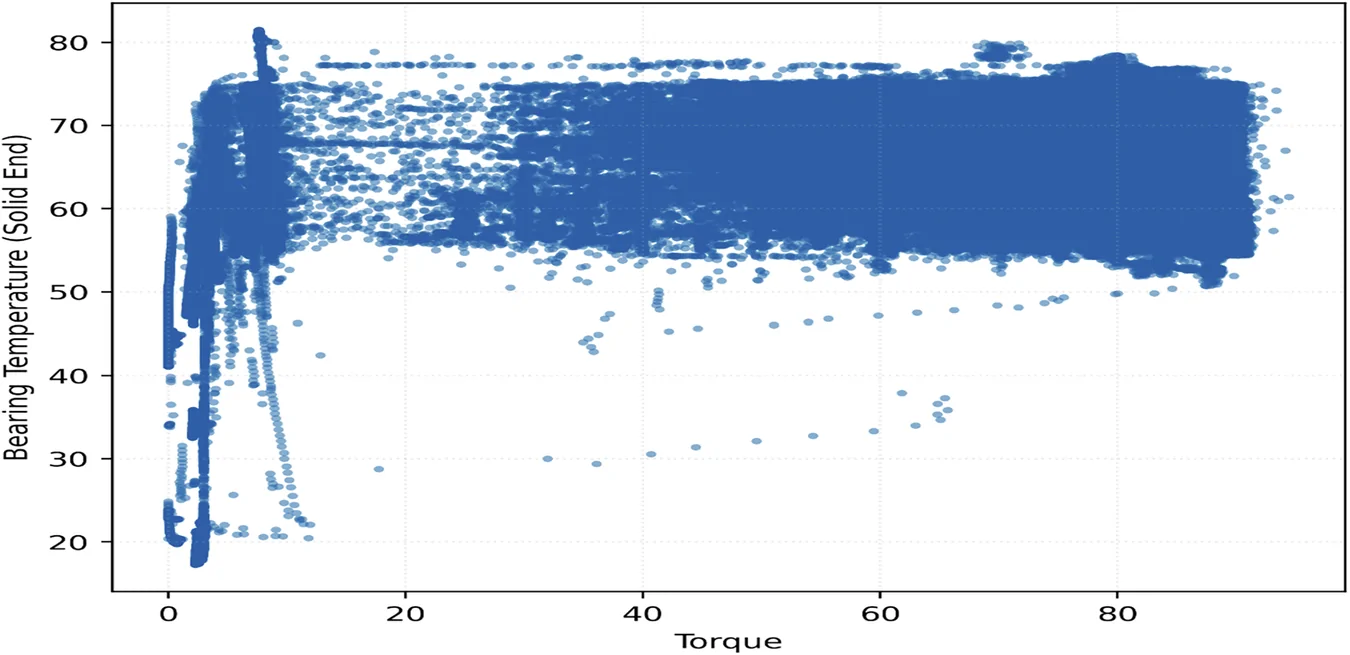
Scatter plot of torque versus solid-end bearing temperature (n = 482,506 observations). Positive association (r = 0.367) reflects frictional heat generation under mechanical load, consistent with the physical coupling analysis in Section 4.4.

The scatter plot between torque and solid-end bearing temperature further confirms a positive relationship between mechanical load and thermal response, indicating that higher torque levels are associated with increased bearing temperatures, as shown (Fig. 12).

#### Comparative interpretability, validity, and full validation summary

Altogether, the set of comparisons undertaken using external standards, public benchmarks, internal operational conditions, physical coupling dynamics, as well as noise versus stability analysis, ensures a full validation of the Sulzer centrifuge data set as a whole. The magnitudes of the data set’s vibrational values remain well within internationally accepted ISO 10816/20816 limits, thus verifying that the observed mechanics during the yearlong period amount to safe, stable, and acceptable industry standards of operation. Comparisons undertaken using standard laboratory data sets, including NASA IMS, PRONOSTIA, XJTU-SY, and FEMTO-ST, accentuate the benefit of this data set, bearing in mind that it is a long-term, multi-modal, real-world data set, which does not include environmental conditions observed in a laboratory-controlled scenario.

Comparisons within the data set itself, for different seasons, loading, and maintenance cycles, have uncovered consistent patterns of data, which correlate well with known machine dynamics, indicating that this data set is anything but randomly variable. The inter-relationships between torque, vibration, and bearing temperature, as established in known machine dynamics studies, also go a long way in ascertaining that this data set is indeed a representation of real machine dynamics in this plant’s turbines. Last but not least, noise analysis in this data set indicates stability in signal fidelity, as would be expected of ABB 800xA DCS in a real-world environment. Taken together, these analyses demonstrate that the dataset is internally consistent, physically coherent, and suitable for exploratory benchmarking in condition monitoring, predictive maintenance, and hybrid physics–ML research. The validation is plant-specific; researchers applying this dataset to other contexts should account for APC-specific operating conditions (Dead Sea industrial environment, APC production cycles) and the DCS-level sampling resolution (1 min), which precludes rotor-dynamic frequency analysis.

## Usage Note

The Sulzer Centrifuge Condition Monitoring Dataset is a unique, openly available benchmarking resource with the potential to drive future innovations in predictive maintenance (PdM) and industrial AI. This dataset is obtained from a regularly functioning centrifuge process at the Arab Potash Company (APC) plant. This real-world dataset provides insight into the production process during steady-state operations as well as during realistic production or environmental variations. This real-world insight applies to the development of machine learning models with predictive potential^21^ at the analytical or prognosis level. It is also particularly applicable for analyzing machine reliability metrics like stress-strength modeling and mean time to degrade or failure^22^ through the association of torques (stress) with corresponding bearing temperatures (strength)^23^.

The dataset is also suitable for creating digital twins or carrying out hybrid simulations with analytical models combined with real-world models to simulate failure modes or operational dynamics^24^. Its multimodal nature makes it suitable for investigations involving sensor fusion modeling, transfer learning, or Explainable Artificial Intelligence with the focus on the paradigm of PHM 4.0^25,26^. By offering the dataset in both Excel and CSV file formats with consistent metadata and with a focus on the principles of FAIR data, this particular dataset provides a reproducible body of truth that is applicable to both academic and industrial benchmarking purposes.

## Data Availability

The dataset used in this study — CFG_42_EDS_Data.xlsx — is publicly available via the Zenodo repository at: https://doi.org/10.5281/zenodo.17885723. The repository provides open, anonymous access to the complete dataset, including all sensor measurements, metadata, and associated documentation required to reproduce the analyses presented in this paper.

## Code availability

All analysis and visualisation were performed using a single Python script (CFG42_Technical_Validation.py). “https://zenodo.org/records/19891670”.
